# Mast cells-intestinal cancer cells crosstalk is mediated by TNF-alpha and sustained by the IL-33/ST2 axis

**DOI:** 10.1007/s00262-025-04054-8

**Published:** 2025-05-15

**Authors:** Chiara Dal Secco, Silvia Tonon, Caterina Trevisan, Eleonora Martinis, Viviana Valeri, Marta Codrich, Gianluca Tell, Barbara Frossi, Carlo E. M. Pucillo

**Affiliations:** 1 Immunology Section, Department of Medicine, University of Udine, Udine, Italy; 2 Molecular Biology Section, Department of Medicine, University of Udine, Udine, Italy

**Keywords:** Mast cells, Organoids, Co-cultures, TNF-α, IL-33, Intestinal differentiation and architecture

## Abstract

It is common knowledge that mast cells (MCs) exert different roles in the gastrointestinal tract, from the maintenance of homeostasis to the onset and propagation of different gut diseases such as food allergies, infections, inflammation, and cancer. However, the mechanisms through which MCs dialog and influence the intestinal tissue are not completely known. To get insight into the bidirectional crosstalk between MCs and the intestinal microenvironment, both in homeostatic and pathological settings, colon organoids from intestinal epithelium of healthy mice and adenomas from AOM/DSS-treated mice have been exploited and co-cultured with MCs. The influence of MCs on organoid architecture and the effect of healthy and tumoral organoids on the phenotype and responsiveness of MCs have been addressed. We observed that MCs interact with intestinal organoids and contribute to the differentiation of healthy organoids by upregulating the expression of mucin-2, chromogranin A, cadherin-1, and claudin 4. On the contrary, in co-culture with tumoral organoids a decrease in cell proliferation, chromogranin A, and lysozyme expression was observed. Tumoral organoids have been shown to activate MCs via the IL-33/ST2 axis leading to increased release of TNF-α which in turn was responsible for the observed effects on tumoral organoids. Our results indicate that MCs are important mediators of intestinal tissue homeostasis and that a different environment can shape and direct MCs toward the dampening or propagation of the inflammatory response. Ultimately, our MC-organoid co-cultures represent a valid in vitro tool to investigate the role of MCs in the gut.

**Supplementary Information:**

The online version contains supplementary material available at 10.1007/s00262-025-04054-8.

## Introduction

Mast cells (MCs) are long-living innate immune cells widely distributed in mucosal and connective tissues at the interface with the external environment. Tissue-specific MCs display differences in their granule content, cytokine expression pattern, and tissue-specific receptors providing context-related functions. Of note, the MC has been described as one of the most plastic cells of the immune system [1]. The gastrointestinal (GI) tract comprises the largest population of MCs in the body, as they represent 2–3% of the immune cell pool in the lamina propria, albeit they can be found also in the muscular and serous layers that harbor nerve and sensory fibers [2]. It is common knowledge that MCs exert different roles in the GI tract, from maintaining homeostatic conditions to the onset and propagation of different GI diseases such as food allergies, infections, inflammatory diseases, and cancer. Indeed, MCs harbor a broad quantity of different preformed and de novo synthesized mediators that can exert a large variety of functions [2] from influencing the intestine blood flow, peristalsis, and mucosal secretion to mediating the crosstalk with adaptive immune cells [3, 4]. Changes in the number of MCs are a common trait of all GI disorders and are often associated with other gastrointestinal alterations, including architectural changes, such as the broadening and flattening of *villi* in the small bowel, and changes in crypt size, shape, and space in the colon. Most of these evidences are correlated with an increased number of MCs, but their role in intestinal tissue development remains mostly unknown [5, 6].

Similarly, their role in the development of colorectal cancer (CRC) is not fully understood and is still under debate. Several studies reported an increase in the number of MCs in the colon of patients affected by intestinal neoplasia, where MCs can be activated and release their pre-stored mediators which, in turn, can affect the integrity of the barriers favoring carcinogenesis [7–10]. However, despite evidence supporting a pro-tumoral role for MCs, other works documented a correlation between high MC density and better clinical outcomes [11, 12]. Difficulties in identifying and isolating intestinal MCs precluded for a long period to decipher their roles in the pathogenesis of CRC, and evidence of their direct effect is still lacking. In this scenario, the use of organoid technology enables the development of an in vitro model that allows the study of these issues. In the past decades, great efforts have been made to develop organoid technology for the study of tissue development, genetic disorders, infection, and cancer [13–15].

In the present work, we took advantage of organoid technology [13, 16] to establish co-cultures of bone marrow-derived mast cells (BMMCs) and organoids obtained from the colon of healthy mice or adenomas derived from AOM/DSS-treated mice that developed inflammation-driven colorectal cancer. This in vitro model allowed us to investigate the bidirectional crosstalk between terminally differentiated BMMCs and the intestinal microenvironment, both in health and in CRC. The effect of BMMCs on the organoid architecture as well as the effect of different organoid types on the phenotype and responsiveness of BMMCs have been addressed.

## Materials and methods

### Mice models

Female 4–8-week-old C57BL/6 mice were purchased from Envigo (Netherlands) and maintained at the animal facility of the Department of Medicine of the University of Udine (Italy). All animal experiments were performed in accordance with institutional guidelines and national law. Colitis-associated colon cancer was induced with an intraperitoneal injection of azoxymethane (AOM, Sigma-Aldrich, 10 mg/kg body weight) in combination with 3 cycles of 2.5% dextran sulfate sodium salt (DSS, MP Biomedicals; MW 36.000–50.000) in drinking water, followed by 14 days of recovery with normal drinking water on 8-week-old mice.

### Mouse organoids and co-cultures with BMMCs

Co-culture experiments between tumoral organoids (TO) or healthy organoids (HO) and 99% pure bone marrow-derived mast cells (BMMCs), as shown in Figure S2, were maintained in ENR medium (supplemental methods) at a ratio of 100.000 BMMCs/Matrigel dome for different timing, as indicated in the figure legends. For separate analysis of organoids or BMMCs, BMMCs were mechanically isolated by gentle pipetting and medium recovery. Several washes with warm PBS were carried out to collect any remaining BMMCs. Organoids were harvested using cell recovery solution (Corning) to dissolve the Matrigel, followed by disaggregation with TryPLE Express (Gibco). Where indicated, co-cultures were performed in presence of 5 µg/ml of anti-ST-2 blocking antibody (Bio-Techne, clone 245,707) or 10µg/ml of TNF-α antibody (Miltenyi, clone MP6-XT22). Detailed information on BMMCs and organoids generation can be found in supplemental information.

### Proliferation assays

After 48 h of co-culture, BMMCs were removed and the proliferation rate of organoids was evaluated by using CellTiter-Glo® Promega Kit according to manufacturer’s instructions.

The proliferation rate of MC38-GFP cells (kindly gifted by Dr. Annalisa Capobianco, I.R.C.C.S. Ospedale San Raffaele, Milano) was assessed by in-cell western blot through the quantification of GFP inside cells directly on the plate, by using anti-GFP mAb (clone GF28R, Invitrogen) and secondary mAb anti-mIgG (H + L) CF^TM^750 (Sigma-Aldrich). The fluorescence was detected with Odissey^R^DLx (LICORbio).

### Flow cytometric analysis

For surface staining of BMMCs or organoids, single-cell suspensions were stained with desired antibodies for 15 min at 4 °C, then washed in PBS + 2% FBS. All samples were acquired with the Attune NxT flow cytometer (Thermo Fisher) and analyzed with the FlowJo software. All antibodies used in flow cytometry are listed in Table S1.

In some experiments, BMMCs were stained with CFSE (Invitrogen) before being added to the organoid cultures, harvested after 48 h of co-culture, and subjected to cytofluorimetric analysis.

For Ki67 staining, organoids were harvested after 48 h of co-culture with BMMCs using the cell recovery solution (Corning), to dissolve the Matrigel, and disaggregated with TryPle Express (Gibco). Briefly, the single-cell suspension was labeled with L/D and cells were fixed by adding drop by drop 3 ml of 70% ethanol under continuous vortexing. Cells were then incubated for 1 h at −20 °C and washed three times with PBS before staining with anti-Ki67 monoclonal antibody (BioLegend) following manufacturer’s instructions. Data were analyzed with FlowJo™ v10.

### RNA extraction and real-time PCR analyses

BMMCs recovered from the supernatant of the cell culture or organoids adequately released from Matrigel were lysed with TRI reagent (Sigma-Merck), and total RNA was extracted according to manufacturer’s instructions. Total RNA was retro-transcribed with the SensiFAST™ cDNA Synthesis kit (Bioline). qPCR analyses were performed with SYBR Green (BioRad) using a BioRad CFX96 real-time PCR detection system. Target gene expression was quantified with the ΔΔCt method using *g3pdh* as a normalizer gene. All primers used are listed in Table S2.

### ELISA assay

Quantification of TNF-α and IL-33 mediators in culture supernatants was performed using specific ELISA kits (eBiosciences) following manufacturer’s instructions.

### Statistical analysis

Experimental data are shown as mean ± standard error of mean (SD). The unpaired or paired Student’s t-test and the one-way or two-way ANOVA (GraphPad Prism 10 Software, La Jolla, CS, USA) were used to assess statistical significance. A confidence level of 95% was used. * = *p* < 0.05, ** = *p* < 0.01, *** = *p* < 0.001.

## Results

### Setup and characterization of intestinal organoids-BMMCs co-cultures: healthy versus pathological organoids differently affect the biology of MCs

To investigate the interaction between intestinal epithelial cells and MCs, we first generated and characterized intestinal organoids from the healthy colon (HO, healthy organoids) and from adenomas (TO, tumoral organoids) of mice treated with azoxymethane (AOM)/dextran sodium sulfate (DSS), as shown in Supplemental Figure S1A-B. TO highly expressed *lgr5* (intestinal stemness marker) and *lyz1* (marker of Paneth cells, normally absent in the colon) while HO had higher expression of differentiation markers, namely *cdh1* (major component of *adherens junctions*), *muc2* (marker for Goblet cells), and *chgA* (marker for enteroendocrine cells) (Supplemental Fig. S1C). We then set up a co-culture system in which BMMCs were placed together with intestinal organoids embedded in Matrigel (Supplemental Fig. S3A). BMMCs were stained with a cell tracker dye (CFSE) before adding them to organoids culture. As shown in Supplemental Fig. S3B, some BMMCs were able to penetrate the Matrigel dome, reached the organoids, and interacted with them at the basolateral side, thus placing themselves in a position that physiologically would constitute the lamina propria where they reside in vivo [17, 18]. In our experiments, we analyzed BMMCs outside the Matrigel dome (Supplemental Fig. S3C).

**Fig. 1:**
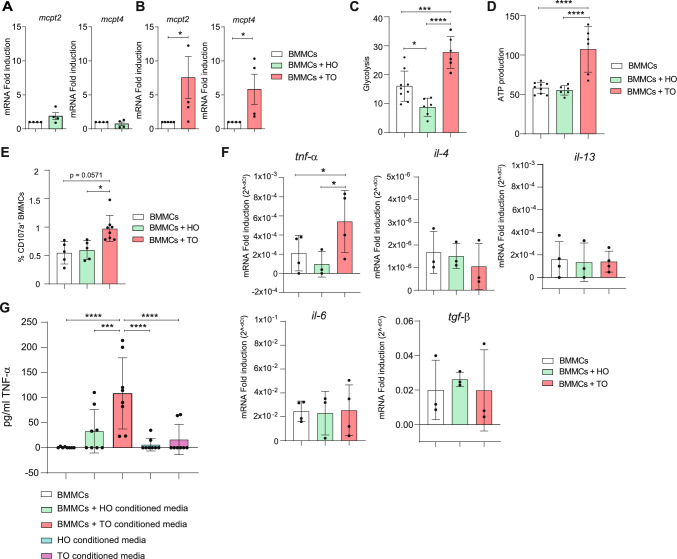
Healthy and tumoral organoids differently affect the behavior of MCs. (**A-B**) qPCR analysis of *mcpt2* and *mcpt4* on BMMCs alone and co-cultured with HO (**A**) or TO (**B**) for 48 h. (**C**) Glycolysis and (**D**) ATP production evaluation in BMMCs alone and co-cultured with HO or TO. (**E**) Percentage of CD107a^+^ expressing BMMCs alone and co-cultured for 24 h with HO or TO. (**F**) qPCR analysis of *tnf-α, il-4, il-13, il-6*, and *tgf-β* on BMMCs alone and co-cultured with HO or TO organoids for 4 h. (**G**) TNF-α quantified by ELISA in supernatant from BMMCs cultured in normal medium or cultured in organoid-derived conditioned media and from organoids conditioned media. Data are expressed as mean + SD from at least n = 3–5 experiments. Statistical analysis was performed with paired Student t-test or with one-way ANOVA with Dunnet correction (* = *p* < 0.05 ** = *p* < 0.01; *** = *p* < 0.001)

First, we studied whether the microenvironment recreated in vitro by means of the organoid cultures could influence the state of activation and phenotype of BMMCs. Hence, the phenotype of BMMCs was evaluated by analyzing the gene expression level of specific proteases expressed by MCs residing in the connective tissue (*mcpt4*) or the mucosa (*mcpt2*). As shown in Fig. 1A, the expression of both *mcpt2* and *mcpt4* genes was unchanged in BMMCs cultured with HO compared with BMMCs alone, while co-culture of BMMCs with TO induced an important increase of both *mcpt4* and *mcpt2* expression (Fig. 1B). Altogether, these data suggest that BMMCs co-cultured with HO maintain the same phenotype, while, in the presence of TO, BMMCs upregulate levels of both *mcpt4* and *mcpt2,* thus promoting a phenotype likely associated with enhanced capability to produce broad-spectrum proteases [19].

Next, the bioenergetic profile of BMMCs was assessed. The analysis revealed that glycolysis was significantly higher in BMMCs maintained in co-culture with TO than with healthy ones (Fig. 1C). Similarly, ATP production increased only in BMMCs that were in presence of TO (Fig. 1D). These results suggest that MCs, when co-cultured with TO, are more metabolically active as they engage more glycolysis and ATP production, probably to sustain their activation. This was further confirmed by cytofluorimetric analysis of the surface expression of LAMP-1 (CD107a), a well-known marker of MCs exocytosis and activation [20]. As shown in Fig. 1E, BMMCs co-cultured for 24 h with TO upregulated LAMP-1 compared to BMMCs grown in the presence of HO or cultured alone, suggesting the potential of TO to potentiate the activation of MCs and granule release. To gain further insight, some mediators known to be released by BMMCs were analyzed. Among them, the expression of the TNF-α mRNA level was increased in MCs co-cultured with TO compared to HO (Fig. 1F). The effect is limited to TNF-α since no changes in gene expression for IL-4, IL-6, IL-13, and TGF-β have been detected (Fig. 1F). To further sustain the ability of TO to induce the activation of MCs and to promote the release of their cytokines, unstimulated BMMCs were incubated with the conditioned media of HO and TO and tested for cytokines secretion.

Higher amounts of TNF-α were detected in the supernatants of BMMCs incubated for 24 h with the conditioned medium of the organoids, especially TO, compared with BMMCs alone (Fig. 1G).

Taken together, these data demonstrate that BMMCs behave differently when co-cultured with healthy or tumor-derived colon organoids. In particular, TO lead to the activation of BMMCs, thus mimicking the in vivo pathological setting [21].

### MCs influence intestinal architecture and cell differentiation

**Fig. 2:**
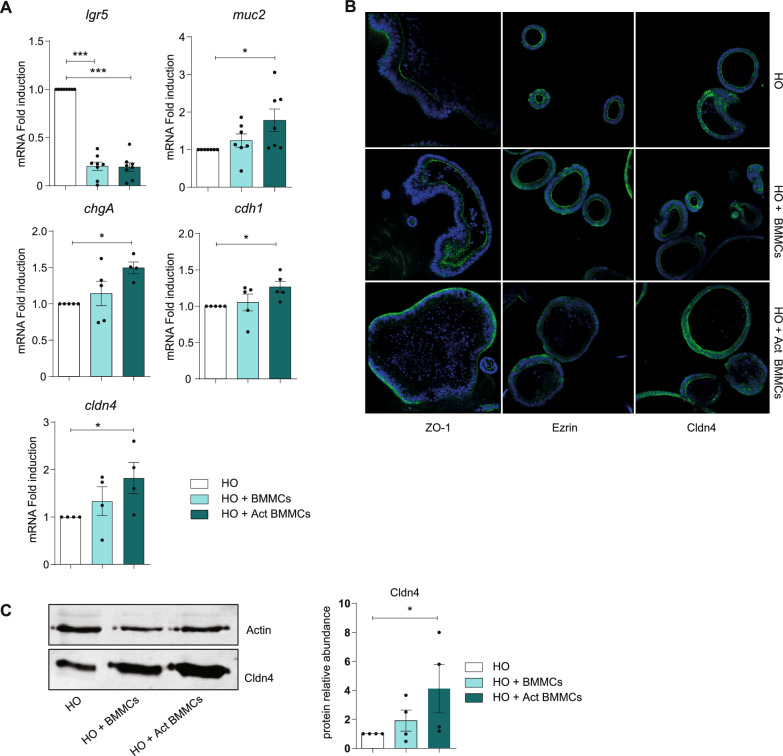
Effect of MCs on the polarization and organization of HO. (**A**) qPCR analysis of *lgr5*, *muc2, chgA, cdh1*, and *cldn4* on HO after 72 h co-culture with resting or IgE/antigen-activated BMMCs (Act BMMCs). (**B**) Immunofluorescence staining of ZO-1, Ezrin, and claudin 4 (all green) in HO co-cultured with resting and activated MCs. Nuclei in blue. (**C**) Western blot analysis of Cldn4 expression in healthy colon organoids cultured alone, and co-culture with resting or activated BMMCs. Right panel shows densitometry analysis calculated over actin expression and normalized versus organoid alone. Data are expressed as mean + SD from n = 3–6 experiments; statistical analysis were performed with one-way ANOVA with Dunnet correction (* = *p* < 0.05 ** = *p* < 0.01; *** = *p* < 0.001)

To investigate the role of MCs in the structural organization of the intestine, HOs were co-cultured for 48 h with resting or IgE/antigen-activated BMMCs. The mere presence of BMMCs, regardless of their activation status, induced a significant downregulation of *lgr5* gene expression, which fell by around 80% (Fig. 2A), suggesting that MCs may be involved in driving the differentiation of major intestinal cell types along the crypt axis. Indeed, in presence of resting and, principally, of IgE/antigen-activated MCs, the mRNA expression levels of the two markers of the secretory lineage *muc2* and *chgA* increased, respectively, by 1 and 0.5 times (Fig. 2A). Moreover, the presence of activated BMMCs seemed to also affect the architectural structure of the organoids, as shown by the altered expression of E-cadherin (*cdh1*), a structural marker, and claudin 4 (*cldn4*), a tight junction expressed throughout all the GI tract, but largely found in the colon (Fig. 2A) [22]. In presence of activated BMMCs, there was an upregulation of *cdh1* and, to a major extent, of c*ldn4*, which is 1.5-fold higher. To deepen these results, immunofluorescence staining (IF) of the co-cultures was performed for the analysis of structural markers such as zonula occludens-1 (ZO-1), a peripheral membrane protein that contributes to the intestinal barrier integrity interacting with the other tight junctions [23] (Fig. 2B, left part), Ezrin, a member of the apical complex that links to the actin cytoskeleton to drive the intestinal epithelium organization [24] (Fig. 2B, middle part), and claudin 4 (Fig. 2B right part). The presence of activated BMMCs caused an altered localization of Ezrin and ZO-1, which were no longer found at the apical level but were delocalized at the basolateral side. Moreover, the immunofluorescence of claudin 4 confirmed the qPCR data, highlighting a *cldn4* gene expression upregulation in the presence of activated BMMCs. The increased amount of claudin 4 protein in the presence of activated BMMCs was further demonstrated by western blot analysis (Fig. 2C).

Taken together, these data suggest that activated MCs influence/impact the organization and constitution of HO by inducing major structural alterations, thus suggesting altered functionality induced by the presence of activated MCs.

### The behavior of tumoral organoids is influenced by MCs

**Fig. 3:**
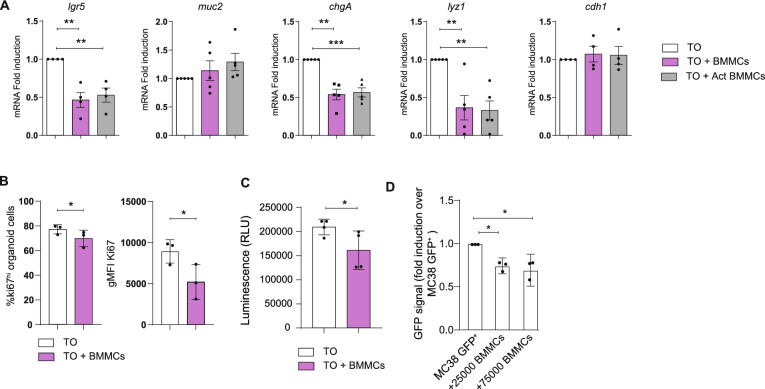
Effect of MCs on the polarization and organization of TO. (**A**) qPCR analysis of *lgr5, muc2, chgA, lyz1*, and *cdh1* on tumoral colon organoids after 72 h of co-culture with resting or IgE/antigen-activated BMMCs. (**B**) Percentage of Ki67^hi^ and Ki67 gMFI of tumoral organoid cells after 48 h of co-culture with resting BMMCs. (**C**) Proliferation of TO through luminescent assay or of (**D**) MC38-GFP cells**,** through evaluation of GFP fluorescence, cultured alone or in the presence of BMMCs. In the case of GFP-MC38 cells, data were normalized over GFP-MC38 cultured alone. Data are expressed as mean + SD from n = 3 experiments. Statistical analysis was performed with paired Student t-test ore one-way ANOVA with Dunnet correction (* = *p* < 0.05 ** = *p* < 0.01; *** = *p* < 0.001)

To assess the role of MCs in the reorganization and differentiation of tumor intestinal tissue, BMMCs were maintained in culture with TO for 48 h. As shown in Fig. 3A, similarly to HO, the presence of BMMCs in the culture caused a significant downregulation of l*gr5* also in TO, which was reduced by 50%. This observation is further supported by bioinformatic analysis on human samples of CRC (TCGA), where MCs abundance negatively correlates with *lgr5* expression in the early stage of tumor development (Supplemental Fig. S5). No changes in *cdh1* and *muc2* were detected (Fig. 3A). Surprisingly, the presence of BMMCs led to a strong downregulation of *chgA*, which drops by 50% (Fig. 3A), inducing an opposite effect compared to the one observed in HO (Fig. 2A). Moreover, since Paneth cells are not usually present in the healthy colon, but can be present in a tumoral context [25, 26], the variation of lysozyme expression (*lyz1*) was also evaluated. As shown before, the expression of *lyz1* mRNA was detectable in TO as compared to its healthy counterpart (Supplemental Fig. S1): In the presence of BMMCs, regardless of their activation state, a significant reduction of *lyz1* mRNA expression was observed (Fig. 3A).

The downregulation of *lgr5, chgA,* and *lyz1* was accompanied by a restriction in cell proliferation (Fig. 3B), where Ki67 expression on TO was evaluated by cytofluorimetric analysis, and in Fig. 3C, where the viability of organoids alone or co-cultured with BMMCs was tested with Cell Titer Glow. To further confirm the effect of MCs on intestinal cell proliferation, the GFP-expressing MC38 colon cancer cell line (MC38-GFP) was cultured for 48 h in the presence or absence of BMMCs at two different BMMCs number ratios. The intensity of the GFP was measured and used as an indicator of cell proliferation. As depicted in Fig. 3D, in the presence of BMMCs, a reduced proliferation of MC38-GFP cells was detected supporting the results observed with TO.

Together, our results suggest that in TO, with respect to HO, MCs exert different effects, thus playing different roles depending on the intestinal microenvironmental clues. In this context, MCs seem to have a positive effect in limiting tumoral cell proliferation.

### MC-released TNF-α influences tumoral organoid status

In peripheral tissues, MCs are an important source of TNF-α, given their ability to release both pre-stored and newly formed TNF-α [27]. Nowadays, increasing evidence indicates that TNF-α is not only detrimental but can also play an important role in health by maintaining homeostasis in the gut [28, 29].

**Fig. 4:**
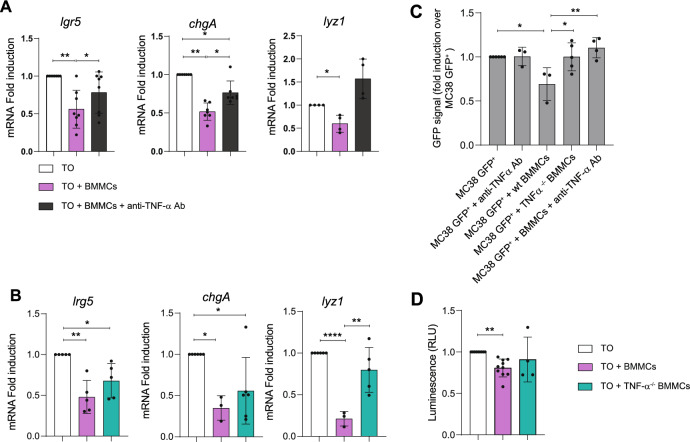
Blocking MC-derived TNF-α attenuates the effects produced on TO. (**A**) qPCR analysis of *lgr5, chgA*, and *lyz1* in TO after 72 h of co-culture with BMMCs in the presence or absence of TNF-α blocking antibody. (**B**) qPCR analysis of *lgr5*, *chgA*, and *lyz1* on TO after 72 h co-culture with wild type (wt) or TNF-α^−/−^ BMMCs. (**C**) Proliferation rates of GFP-MC38 cultured alone or in presence of TNF-α blocking antibody, wt BMMCs, TNF-α^−/−^ BMMCs, or wt BMMCs together with TNF-α blocking ab. (**D**) Proliferation rates of TO cultured alone or in presence TNF-α ^−/−^ BMMCs or wt BMMCs. Data are expressed as mean + SD from n = 3–8 experiments. Statistical analysis was performed with one-way ANOVA with Dunnet correction (* = *p* < 0.05 ** = *p* < 0.01; *** = *p* < 0.001)

As shown in Fig. 1J, we observed that TO-conditioned media was able to induce considerable release of TNF-α by BMMCs. Therefore, we performed BMMCs-organoids co-cultures in the presence/absence of a specific blocking antibody against TNF-α. As demonstrated by data in Fig. 4A, in the presence of the TNF-α blocking antibody the expression of *lgr5*, *chgA*, and *lyz1* was maintained. The regulation of *lgr5*, *chgA*, and *lyz1* was further investigated by co-culturing TO with TNF-α^−/−^ BMMCs (Fig. 4B). *lyz1* downregulation strongly depended on TNF-α produced by BMMCs, while *lgr5*, *chgA* downregulation was still partially present and might depend also on autocrine TNF-α produced by TO. As further evidence, we also analyzed MC38 proliferation: The reduction of GFP-MC38 proliferation in co-culture with BMMCs was attenuated in the presence of TNF-α blocking ab or TNF-α-deficient BMMCs (Fig. 4C), thus confirming that the effects showed in Fig. 3 are mediated by TNF-α. Moreover, the proliferation of TO was not reduced in presence of TNF-α-deficient BMMCs (Fig. 4D). Collectively, these data show that TNF-α is a major mediator in the regulation of TO proliferation and of *lgr5*, *chgA*, and *lyz1* expression in TO.

### The role of ST2/IL-33 axis in MC-tumoral organoids interaction

**Fig. 5:**
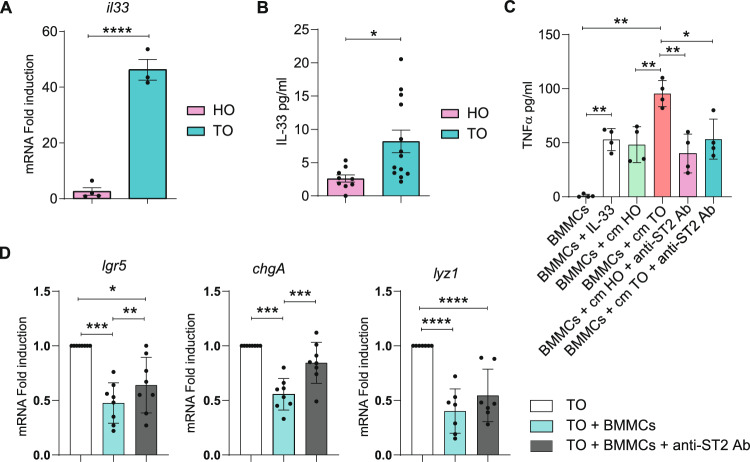
The ST2/IL-33 axis plays a role in MCs-organoids interaction. (**A**) qPCR analysis of *Il-33* expression in HO and TO. (**B**) Quantification of soluble IL-33 released from HO or TO in the culture medium was determined by ELISA. (**C**) Quantification by ELISA of TNF-α secreted by BMMCs alone, after incubation with 50 ng/ml of recombinant IL-33 and after cell culture in organoid-conditioned media (cm) in the presence/absence of ST2-blocking antibody. (**D**) qPCR analysis of *lgr5*, *chgA*, and *lyz1* expression on TO organoids after 72 h of co-culture with BMMC in the presence or absence of ST2-blocking antibody. Data are expressed as mean + SD from n = 3–8 experiments. Statistical analysis was performed with Student t-test or one-way ANOVA with Dunnet correction (* = *p* < 0.05 ** = *p* < 0.01; *** = *p* < 0.001)

Upon barrier disruption, IL-33 is known to recruit and activate innate immune cells toward a type 2 inflammatory response with the primary goal of tissue regeneration [30, 31] and to activate intraepithelial lymphocytes, basophils, eosinophils, and MCs [32]. Therefore, we asked whether intestinal organoids expressed IL-33, and if so, whether organoid-derived IL-33 was conceivable responsible for the activation of BMMCs and TNF-α secretion. In our system intestinal organoids, both HO and TO expressed and secreted IL-33 (Fig. 5A-B) with TO having a 50-times fold increase of *Il-33* gene expression compared to HO. As expected, stimulation of resting BMMCs with recombinant IL-33 induced the release of TNF-α (Fig. 5C). We obtained TNF-α release from BMMCs also by stimulating them with the conditioned media of both HO and TO, with higher effect produced when using TO supernatants (Fig. 5C).

Notably, the addition of the ST2-blocking antibody, which prevents the binding of IL-33 on target cells, reduced the release of TNF-a by BMMCs in culture with TO, but not HO, derived conditioned medium (Fig. 5C). It is possible to speculate that HO induce basal release of TNF-α from MCs via an IL-33-independent mechanism, while, in tumor conditions, the IL-33 present in the co-culture can induce a greater release of TNF-α which may be responsible for the effects observed on TO.

We next performed co-culture experiments in the presence of the ST2-blocking antibody to assess whether the IL-33-dependent activation of MCs was responsible for influencing the expression of intestinal markers by qPCR (Fig. 5D). We observed a statistically relevant restoration of *lgr5* and *chgA lyz1* (and a similar trend for *lyz1*), expression levels when the ST2/IL-33 axis was blocked which indicates the relevancy of a micromilieu enriched of IL-33 in modulating the MCs/intestinal organoids crosstalk.

## Discussion

The plasticity of MCs is of major importance in the gut where encountering external environment and pathogenic stimuli is more likely to happen [17]. In the intestine, MCs are a rare, interspersed cell population, which makes it difficult to properly analyze their role in both the regulation of physiological processes and their involvement in tumor initiation or progression. For this reason, we set up an in vitro co-culture system using intestinal organoids. The great advantage of the organoid model, in this work, is that it sheds light on the role of MCs in both healthy and pathological gut contexts, allowing the 3D architecture and cellular composition of the gut to be studied [33]. In the present study, we mainly used the co-culture system of BMMCs with organoids from healthy colon or derived from adenomas of AOM/DSS-treated mice. The AOM/DSS model resembles the human adenomas of colitis associated-CRC and, in particular, our organoids present a mutation in the exon 3 of *ctnnb1* gene (with wild-type *Kras*, data not shown) leading to an aberrant Wnt pathway activation [34], a signaling pathway whose alteration is fundamental for the initiation and progression of CRC [35]. Indeed, our tumoral organoids harbor a mutation in β-catenin and still express several markers of differentiated intestinal cells albeit at lower levels respect to organoids obtained from healthy tissue (Supplemental Fig. S1C). We can therefore consider our model as an early stage of CRC that can allow us to investigate the roles of MCs in the onset of CRC. Indeed, the analysis of the mutations present in our TO is equivalent to those found in human tumors at the same stage of development [36].

Common understanding indicates that MCs can be both, directly and indirectly, involved in various intestinal pathologies, not least CRC progression. However, published data on the role of MCs in tumor progression are still contradictory [10]. One possible explanation relies on the intrinsic plastic nature of the MCs: They sense the surrounding microenvironment shaping and adapting their response accordingly [27, 37]. Additionally, it should be taken into consideration the time frame in which MCs act on the tumor since these cells could have opposing roles during the onset or the progression of the pathology.

We herein demonstrated that BMMCs interact with the basolateral side of intestinal organoids, namely the *lamina propria* side, where MCs are normally found in vivo. We further demonstrated that MCs are attracted by the organoids and can directly reach the epithelium by actively moving inside the Matrigel (Fig. S3B). To our knowledge, no differences in the number of MCs actively interacting with healthy rather than tumoral organoids have been reported to date. However, it has been shown that MCs, even in the absence of direct contact, generate a different response in healthy or pathological contexts, modulating the expression of structural parameters and cell viability. As a matter of fact, we here have pointed out how the microenvironment indirectly exerts an effect on the polarization of the MCs response, which in turn induces different outcome on the organoid depending on the setting (e.g., HO or TO). BMMCs, normally used for in vitro experiments, express an intermediate phenotype between mucosal and connective tissue MCs. We demonstrated that BMMCs co-cultured with healthy colon organoids maintain their immature phenotype. Differently, when co-cultured with TO, they significantly upregulate the expression of both *mcpt2* and *mcpt4*, suggesting that BMMCs received strong activating stimuli from the tumor and respond increasing the transcription of both proteases analyzed. These proteases can play a relevant role in tissue remodeling during tumorigenesis [38]. The evidence that MCs accumulate and are activated in the tumor microenvironment is well-established [7, 8]. As demonstrated by the upregulation of CD107a, the co-culture with TO resulted in BMMC activation and granule release. In this scenario, we hypothesized that tumor-derived IL-33 could participate in the strong activation of BMMCs. As the literature reports, adenoma/CRC cells express high levels of IL-33 mRNA [39], and our TO secrete higher levels of IL-33 compared to the healthy colon, supporting the strong validity of the TO model. We were able to demonstrate that a functional IL-33/ST2 axis was required to maintain high levels of TNF-α production and release by BMMCs. Of note, MCs represent the only cell type able to store preformed TNF-α in their granules and, upon exocytosis, can exhibit antitumor activity through direct tumor cell cytotoxicity mediated by TNF-α and reactive oxygen species [40]. Moreover, a transcriptomic analysis performed on MCs incubated with HT-29 spheroids unveils TNFSF14 among the transcripts upregulated in MCs suggesting that the CRC cells can activate the TNF-a pathway [41]. The role of this mediator is crucial in CRC development and produces multiple effects. Saito and collaborators showed that treatment with recombinant TNF-α was sufficient to promote an inflammatory response and decreased intestinal stem cell activity and barrier function. The authors showed that TNF-α given to enteric culture can reduce mRNA level of *lrg5*, *muc2,* and *chgA* [42], an effect that, in part, we reproduced in our conditions: BMMCs induced the modulation of *lgr5* and *chgA* expression through TNF-α release (Fig. 4A-B). Interestingly, as suggested in Fig. 5C, the abrogation of IL-33/ST2 pathway did not completely abolished the TNF-α release, suggesting that other soluble factors are involved. A possible candidate could be the stem cell factor (SCF); in fact, an accumulation of this factor from tumor lysates derived from the AOM/DSS model was reported before. However, the SCF, also present in the lysates from healthy tissue, was not able to induce TNF-α release [43]. Our observation, that a basal level of TNF-α is detectable from the BMMCs/HO co-culture medium, is in line with a recent report that describes how low TNF-α amounts contribute to maintain intestinal homeostasis in healthy conditions [28].

According to our observations, MCs can dampen tumor progression in the early phases of tumor progression. In our model, they promote the downregulation of the Ki67 marker and of *lgr5* and proliferation in TO, all effects mediated by MCs-derived TNF-α. Besides, we hypothesize that TNF-α is released by BMMCs in two different time frames: Pre-stored TNF-α is released immediately, hypothetically by the effect of mediators such as the SCF, then newly formed TNF-α production and release is further sustained by IL-33 stimulation. Indeed, when we blocked the TNF-α pathway in the co-culture, we observed a maintenance of *lgr5*, *lyz1,* and *chgA* expression, while α-ST2 treatment was less incisive on the expression levels of these genes, suggesting that their transcription was already compromised. Indeed, we cannot also exclude that the effect is due to TNF-α produced by organoids themselves that can still abolish the expression of above-mentioned genes.

Taken together these data demonstrate the validity of our BMMCs/intestinal organoids co-culture model which is instrumental to assess molecular and biochemical changes in both MCs and intestinal cells. Bioinformatic analyses from human CRC samples (Supplemental Fig. S2) seem to go in line with some of our results: In the early tumorigenesis phase (T1), more abundance of MCs negatively correlates with the *lrg5* expression on cancer tissue. Even though it did not reach a statistical significance, proliferation and *lyz1* expression of cancer tissue inversely correlated with the density of MCs in the T1 size. These data from human samples sustain our model in which the antitumoral effect of MCs is produced in the first phases of tumor development while the role of MCs in later stages requires further studies. In conclusion, the co-culture system herein described is a valuable model for studying the role of MCs in the early phase of CRC progression and can be used to test new candidate drugs targeting either MCs or CRC organoids.

## Supplementary Information

Below is the link to the electronic supplementary material.

Supplementary file1 (DOCX 2176 KB)

## Data Availability

No datasets were generated or analyzed during the current study.

## References

[1] Dwyer DF, Barrett NA, Austen KF et al (2016) Expression profiling of constitutive mast cells reveals a unique identity within the immune system. Nat Immunol 17:878–887. 10.1038/NI.3445 27135604 10.1038/ni.3445 PMC5045264

[2] Bischoff SC, Krämer S (2007) Human mast cells, bacteria, and intestinal immunity. Immunol Rev 217:329–337. 10.1111/J.1600-065X.2007.00523.X 17498069 10.1111/j.1600-065X.2007.00523.x

[3] Bischoff SC (2009) Physiological and pathophysiological functions of intestinal mast cells. Semin Immunopathol 31:185–205. 10.1007/s00281-009-0165-4 19533134 10.1007/s00281-009-0165-4

[4] Merluzzi S, Frossi B, Gri G et al (2010) Mast cells enhance proliferation of B lymphocytes and drive their differentiation toward IgA-secreting plasma cells. Blood 115:2810–2817. 10.1182/blood-2009-10-250126 20101023 10.1182/blood-2009-10-250126

[5] Groschwitz KR, Ahrens R, Osterfeld H et al (2009) Mast cells regulate homeostatic intestinal epithelial migration and barrier function by a chymase/Mcpt4-dependent mechanism. Proc Natl Acad Sci 106:22381–22386. 10.1073/pnas.0906372106 20018751 10.1073/pnas.0906372106 PMC2799737

[6] Kirsch R, Geboes K, Shepherd NA et al (2008) Systemic mastocytosis involving the gastrointestinal tract: clinicopathologic and molecular study of five cases. Mod Pathol Off J US Can Acad Pathol 21:1508–1516. 10.1038/MODPATHOL.2008.158 10.1038/modpathol.2008.158 18931652

[7] Malfettone A, Silvestris N, Saponaro C et al (2013) High density of tryptase-positive mast cells in human colorectal cancer: a poor prognostic factor related to protease-activated receptor 2 expression. J Cell Mol Med. 10.1111/jcmm.12073 23991686 10.1111/jcmm.12073 PMC3780541

[8] Rigoni A, Colombo MP, Pucillo C (2015) The role of mast cells in molding the tumor microenvironment. Cancer Microenviron 8:167–176. 10.1007/s12307-014-0152-8 25194694 10.1007/s12307-014-0152-8 PMC4715001

[9] Rigoni A, Bongiovanni L, Burocchi A et al (2015) Mast cells infiltrating inflamed or transformed gut alternatively sustain mucosal healing or tumor growth. Can Res 75:3760–3770. 10.1158/0008-5472.CAN-14-3767/651844/AM/MAST-CELLS-INFILTRATING-INFLAMED-OR-TRANSFORMED 10.1158/0008-5472.CAN-14-3767 26206557

[10] Liu X, Li X, Wei H et al (2023) Mast cells in colorectal cancer tumour progression, angiogenesis, and lymphangiogenesis. Front Immunol 14:1209056. 10.3389/fimmu.2023.1209056 37497234 10.3389/fimmu.2023.1209056 PMC10366593

[11] Mehdawi L, Osman J, Topi G, Sjölander A (2016) High tumor mast cell density is associated with longer survival of colon cancer patients. Acta Oncol 55:1434–1442. 10.1080/0284186X.2016.1198493 27355473 10.1080/0284186X.2016.1198493

[12] Nielsen HJ, Hansen U, Christensen IJ et al (1999) Independent prognostic value of eosinophil and mast cell infiltration in colorectal cancer tissue. J Pathol 189:487–495. 10.1002/(SICI)1096-9896(199912)189:4%3c487::AID-PATH484%3e3.0.CO;2-I 10629548 10.1002/(SICI)1096-9896(199912)189:4<487::AID-PATH484>3.0.CO;2-I

[13] Clevers H (2016) Modeling development and disease with organoids. Cell 165:1586–1597. 10.1016/J.CELL.2016.05.082 27315476 10.1016/j.cell.2016.05.082

[14] Drost J, Clevers H (2018) Organoids in cancer research. Nat Rev Cancer 18:407–418. 10.1038/S41568-018-0007-6 29692415 10.1038/s41568-018-0007-6

[15] Fatehullah A, Tan SH, Barker N (2016) Organoids as an in vitro model of human development and disease. Nat Cell Biol 18:246–254. 10.1038/NCB3312 26911908 10.1038/ncb3312

[16] Dutta D, Heo I, Clevers H (2017) Disease modeling in stem cell-derived 3D organoid systems. Trends Mol Med 23:393–410. 10.1016/J.MOLMED.2017.02.007 28341301 10.1016/j.molmed.2017.02.007

[17] Bischoff SC (2007) Role of mast cells in allergic and non-allergic immune responses: comparison of human and murine data. Nat Rev Immunol 7:93–104. 10.1038/nri2018 17259966 10.1038/nri2018

[18] Mion F, D’Inca F, Danelli L et al (2014) Mast cells control the expansion and differentiation of IL-10-competent B cells. J Immunol 193:4568–4579. 10.4049/jimmunol.1302593 25267976 10.4049/jimmunol.1302593

[19] Tauber M, Basso L, Martin J et al (2023) Landscape of mast cell populations across organs in mice and humans. J Exp Med 220:e20230570. 10.1084/jem.20230570 37462672 10.1084/jem.20230570 PMC10354537

[20] Grützkau A, Smorodchenko A, Lippert U et al (2004) LAMP-1 and LAMP-2, but not LAMP-3, are reliable markers for activation-induced secretion of human mast cells. Cytom Part A J Int Soc Anal Cytol 61:62–68. 10.1002/CYTO.A.20068 10.1002/cyto.a.20068 15351990

[21] Molfetta R, Paolini R (2023) The controversial role of intestinal mast cells in colon cancer. Cells 12:459. 10.3390/cells12030459 36766801 10.3390/cells12030459 PMC9914221

[22] Zhu L, Han J, Li L et al (2019) Claudin family participates in the pathogenesis of inflammatory bowel diseases and colitis-associated colorectal cancer. Front Immunol. 10.3389/fimmu.2019.01441 31316506 10.3389/fimmu.2019.01441 PMC6610251

[23] Sturgeon F (2016) Zonulin, a regulator of epithelial and endothelial barrier functions, and its involvement in chronic inflammatory diseases. Tissue Barriers. 10.1080/21688370.2016.1251384 28123927 10.1080/21688370.2016.1251384 PMC5214347

[24] Casaletto JB, Saotome I, Curto M, McClatchey AI (2011) Ezrin-mediated apical integrity is required for intestinal homeostasis. Proc Natl Acad Sci 108(29):11924–11929. 10.1073/pnas.1103418108 21730140 10.1073/pnas.1103418108 PMC3141968

[25] López-Arribillaga E, Yan B, Lobo-Jarne T et al (2021) Accumulation of paneth cells in early colorectal adenomas is associated with beta-catenin signaling and poor patient prognosis. Cells. 10.3390/cells10112928 34831152 10.3390/cells10112928 PMC8616107

[26] Shin JH, Park J, Lim J et al (2024) Metastasis of colon cancer requires Dickkopf-2 to generate cancer cells with Paneth cell properties. eLife. 10.7554/eLife.97279.2 39535280 10.7554/eLife.97279 PMC11560131

[27] De Zuani M, Dal Secco C, Tonon S et al (2022) LPS guides distinct patterns of training and tolerance in mast cells. Front Immunol. 10.3389/fimmu.2022.835348 35251027 10.3389/fimmu.2022.835348 PMC8891506

[28] Reyes EA, Castillo-Azofeifa D, Rispal J et al (2023) Epithelial TNF controls cell differentiation and CFTR activity to maintain intestinal mucin homeostasis. J Clin Invest. 10.1172/JCI163591 37643009 10.1172/JCI163591 PMC10575728

[29] Ruder B, Atreya R, Becker C (2019) Tumour necrosis factor alpha in intestinal homeostasis and gut related diseases. Int J Mol Sci 20:1887. 10.3390/ijms20081887 30995806 10.3390/ijms20081887 PMC6515381

[30] Martin NT, Martin MU (2016) Interleukin 33 is a guardian of barriers and a local alarmin. Nat Immunol 17:122–131. 10.1038/NI.3370 26784265 10.1038/ni.3370

[31] Cohen ES, Scott IC, Majithiya JB et al (2015) Oxidation of the alarmin IL-33 regulates ST2-dependent inflammation. Nat Commun 6(1):1–10. 10.1038/ncomms9327 10.1038/ncomms9327 PMC4579851 26365875

[32] Liew FY, Girard JP, Turnquist HR (2016) Interleukin-33 in health and disease. Nat Rev Immunol 16:676–689. 10.1038/NRI.2016.95 27640624 10.1038/nri.2016.95

[33] Sato T, Vries RG, Snippert HJ et al (2009) Single Lgr5 stem cells build crypt-villus structures in vitro without a mesenchymal niche. Nature 459:262–265. 10.1038/nature07935 19329995 10.1038/nature07935

[34] Gao C, Wang Y, Broaddus R et al (2017) Exon 3 mutations of CTNNB1 drive tumorigenesis: a review. Oncotarget 9:5508–5492 10.18632/oncotarget.23695 PMC5797067 29435196

[35] Nie X, Liu H, Liu L et al (2020) Emerging roles of Wnt ligands in human colorectal cancer. Front Oncol. 10.3389/fonc.2020.01341 32923386 10.3389/fonc.2020.01341 PMC7456893

[36] Schmitt M, Greten FR (2021) The inflammatory pathogenesis of colorectal cancer. Nat Rev Immunol 21:653–667. 10.1038/s41577-021-00534-x 33911231 10.1038/s41577-021-00534-x

[37] Frossi B, Mion F, Sibilano R et al (2018) Is it time for a new classification of mast cells? What do we know about mast cell heterogeneity? Immunol Rev 282:35–46. 10.1111/imr.12636 29431204 10.1111/imr.12636

[38] Atiakshin D, Kostin A, Buchwalow I et al (2022) Protease profile of tumor-associated mast cells in melanoma. Int J Mol Sci 23:8930. 10.3390/ijms23168930 36012196 10.3390/ijms23168930 PMC9408654

[39] Cui G, Yuan A, Pang Z et al (2018) Contribution of IL-33 to the pathogenesis of colorectal cancer. Front Oncol. 10.3389/FONC.2018.00561 30547011 10.3389/fonc.2018.00561 PMC6279916

[40] Majorini MT, Colombo MP, Lecis D (2022) Few, but efficient: the role of mast cells in breast cancer and other solid tumors. Can Res 82:1439–1447. 10.1158/0008-5472.CAN-21-3424 10.1158/0008-5472.CAN-21-3424 PMC9306341 35045983

[41] Yu Y, Blokhuis BR, Garssen J, Redegeld FA (2019) A transcriptomic insight into the impact of colon cancer cells on mast cells. Int J Mol Sci 20:1689. 10.3390/ijms20071689 30987352 10.3390/ijms20071689 PMC6480031

[42] Saito Y, Shimizu M, Iwatsuki K et al (2021) Effect of short-time treatment with TNF-α on stem cell activity and barrier function in enteroids. Cytotechnology 73:669–682. 10.1007/s10616-021-00487-y 34349355 10.1007/s10616-021-00487-y PMC8319277

[43] Molfetta R, Lecce M, Milito ND et al (2023) SCF and IL-33 regulate mouse mast cell phenotypic and functional plasticity supporting a pro-inflammatory microenvironment. Cell Death Dis 14:616. 10.1038/s41419-023-06139-7 37730723 10.1038/s41419-023-06139-7 PMC10511458

