# Supplementary material for: Mast cells-intestinal cancer cells crosstalk is mediated by TNF-alpha and sustained by the IL-33/ST2 axis
Source: Cancer Immunol Immunother. 2025 May 15;74(7):205. doi: 10.1007/s00262-025-04054-8 (PMC12081814; doi:10.1007/s00262-025-04054-8)
Supplement: Supplementary file 1 — Supplementary file1 (DOCX 2176 KB) [file 262_2025_4054_MOESM1_ESM.docx]

**SUPPLEMENTAL INFORMATION**


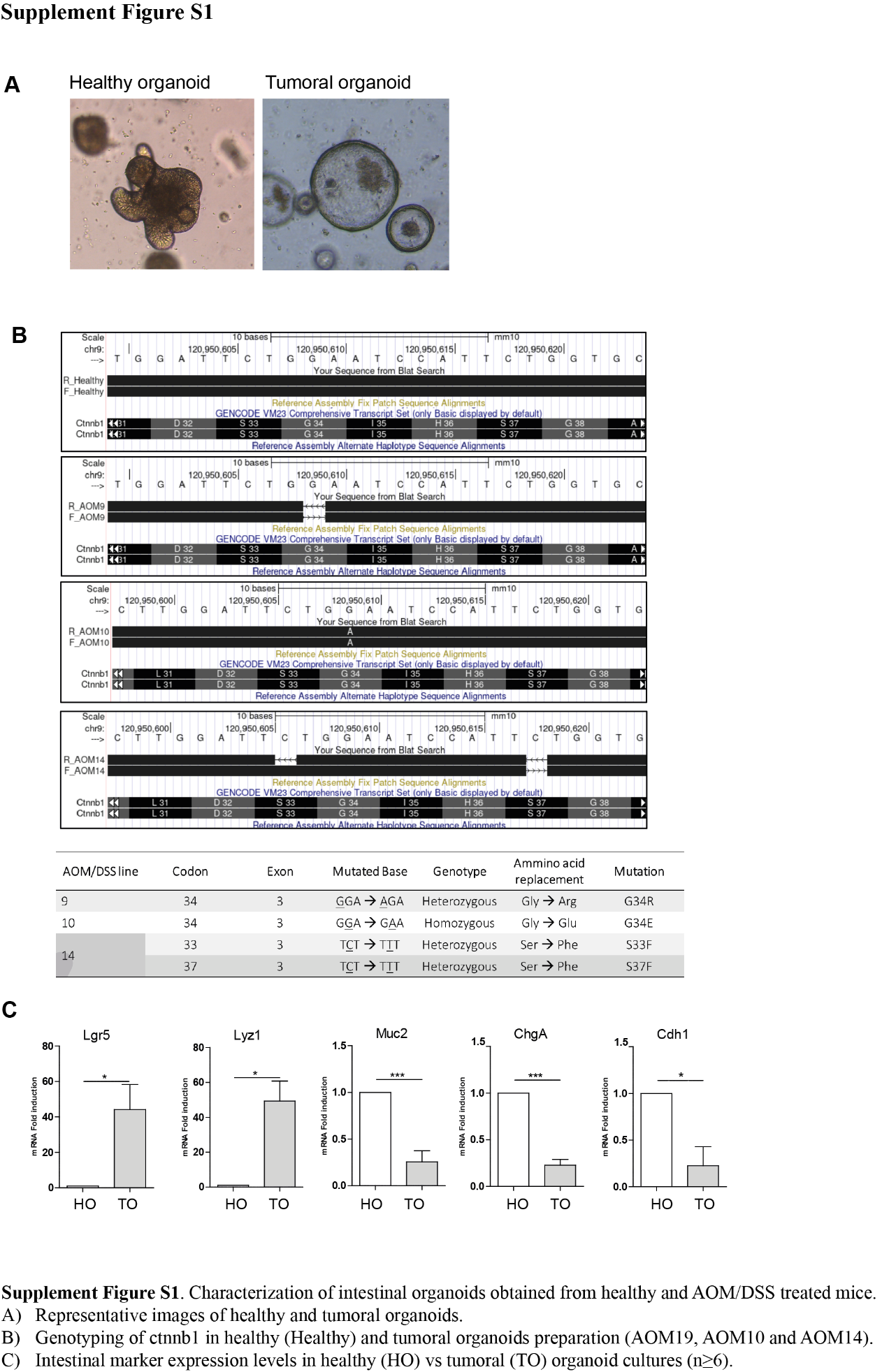


**Supplemental Figure S1. Characterization of intestinal organoids obtained from healthy and AOM/DSS treated mice.** (A) Representative images of healthy and tumoral organoids cultured in ENR. (B) Genotyping of cnntb1 in healthy (Healthy) and tumoral organoids preparation (AOM9, AOM10 and AOM14). (C) Intestinal marker expression levels in healthy (HO) vs tumoral (TO) organoids cultures (n >= 6)

**Supplemental Figure S2**

| **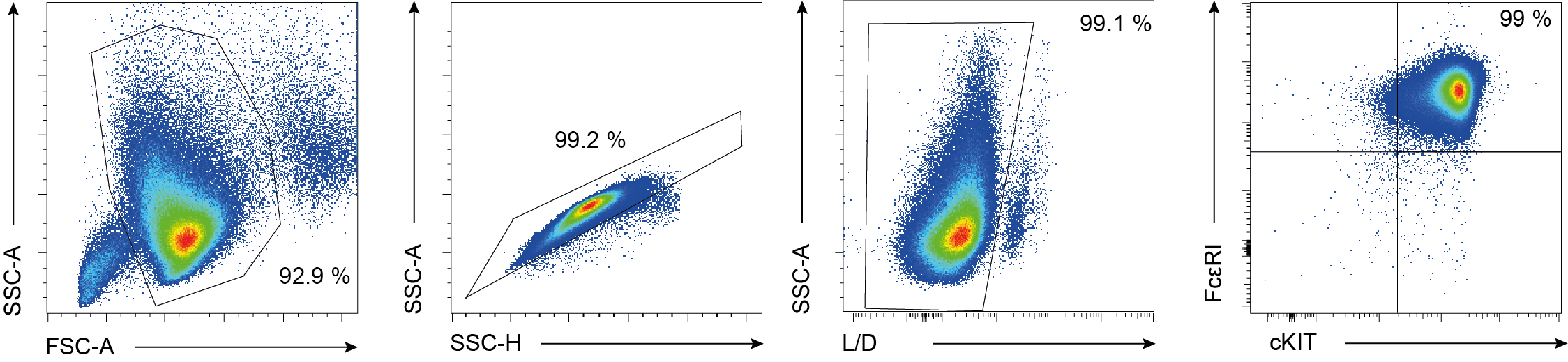** |
| --- |
| **Supplemental Figure S2**. **BMMCs differentiation.** Representative flow cytometry plots of BMMCs culture purity. Among viable cells the 99% of them was double positive for cKIT and FcεRI. |

**Supplemental Figure S3**

| A | 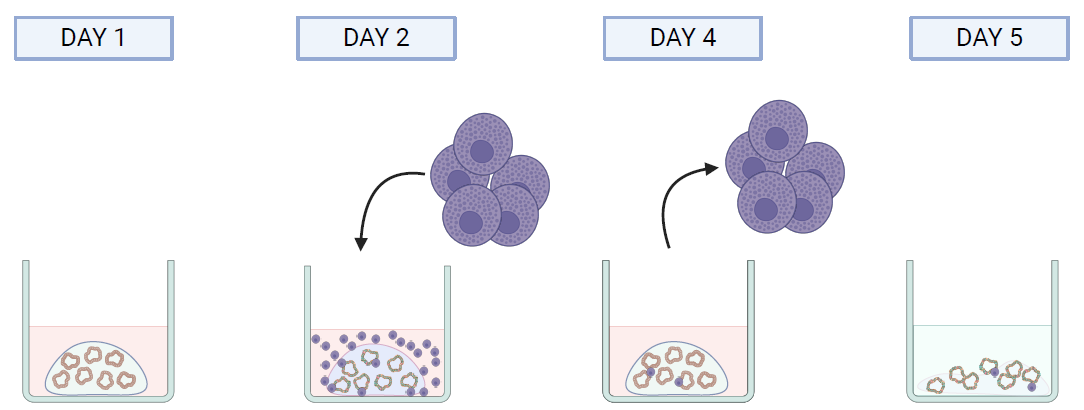 |
| --- | --- |
| B | 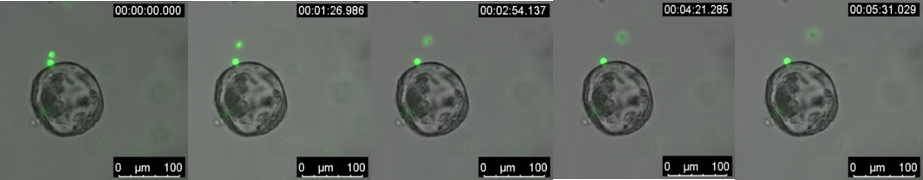 |
| C | 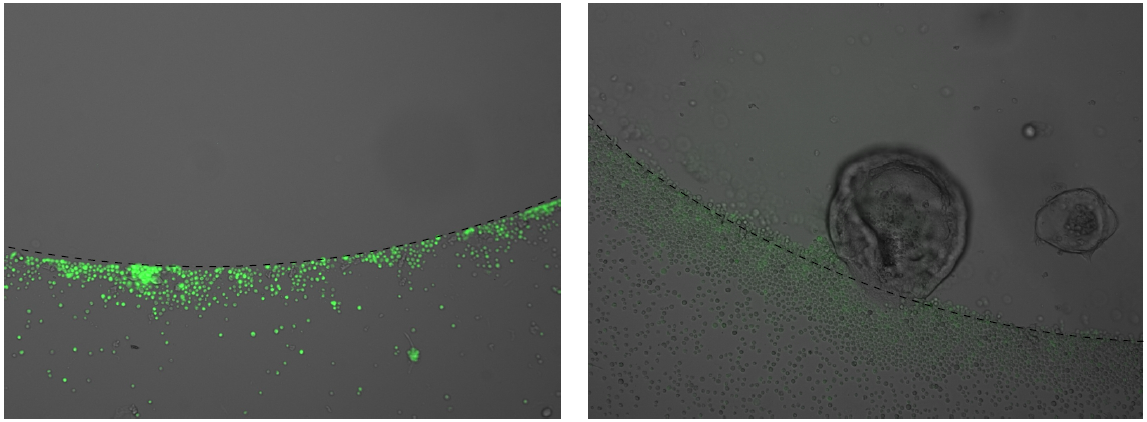 |
| **Supplemental Figure S3. Visualization of BMMCs-organoids interaction. (A)** organoids-BMMCs co-culture. Day1: Organoids were passaged and placed in complete WENR medium for 24h; Day2: BMMCs were resuspended in ENR medium and placed over the organoids containing matrigel domes (ratio of 100.000 cells/matrigel dome); Day4: BMMCs in suspension (not direct organoid-BMMCs interaction) collected for cytofluorimetric and qPCR analysis; Day5: Thorough washing of the well (warm PBS) before matrix disaggregation (Cell Recovery Solution(Corning)) and subsequent collection and analysis of organoids. **(B)** Time-lapse frames of CFSE-labeled BMMCs (green) interacting with intestinal organoid. Images were taken every 10 seconds for a total of 5’39’’. (C) Representative Images CFSE-labelled BMMCs (green) at the edge of an empty Matrigel dome (left panel) and with organoids (right panel) | |

**Supplemental Figure S4**

| A | 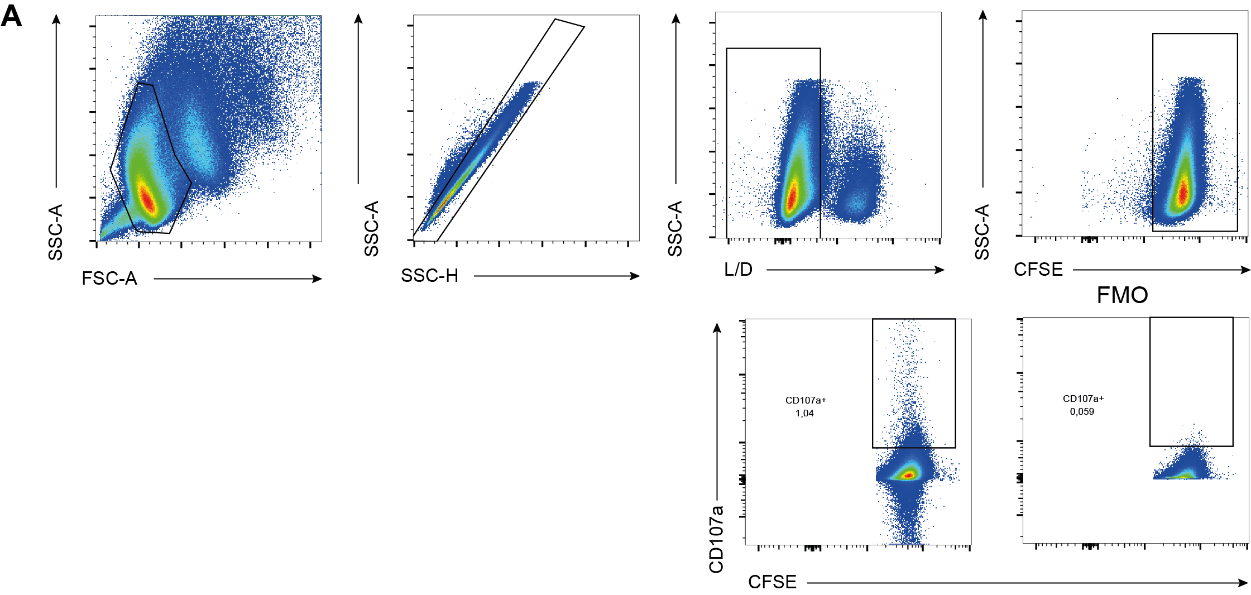 |
| --- | --- |
| B | 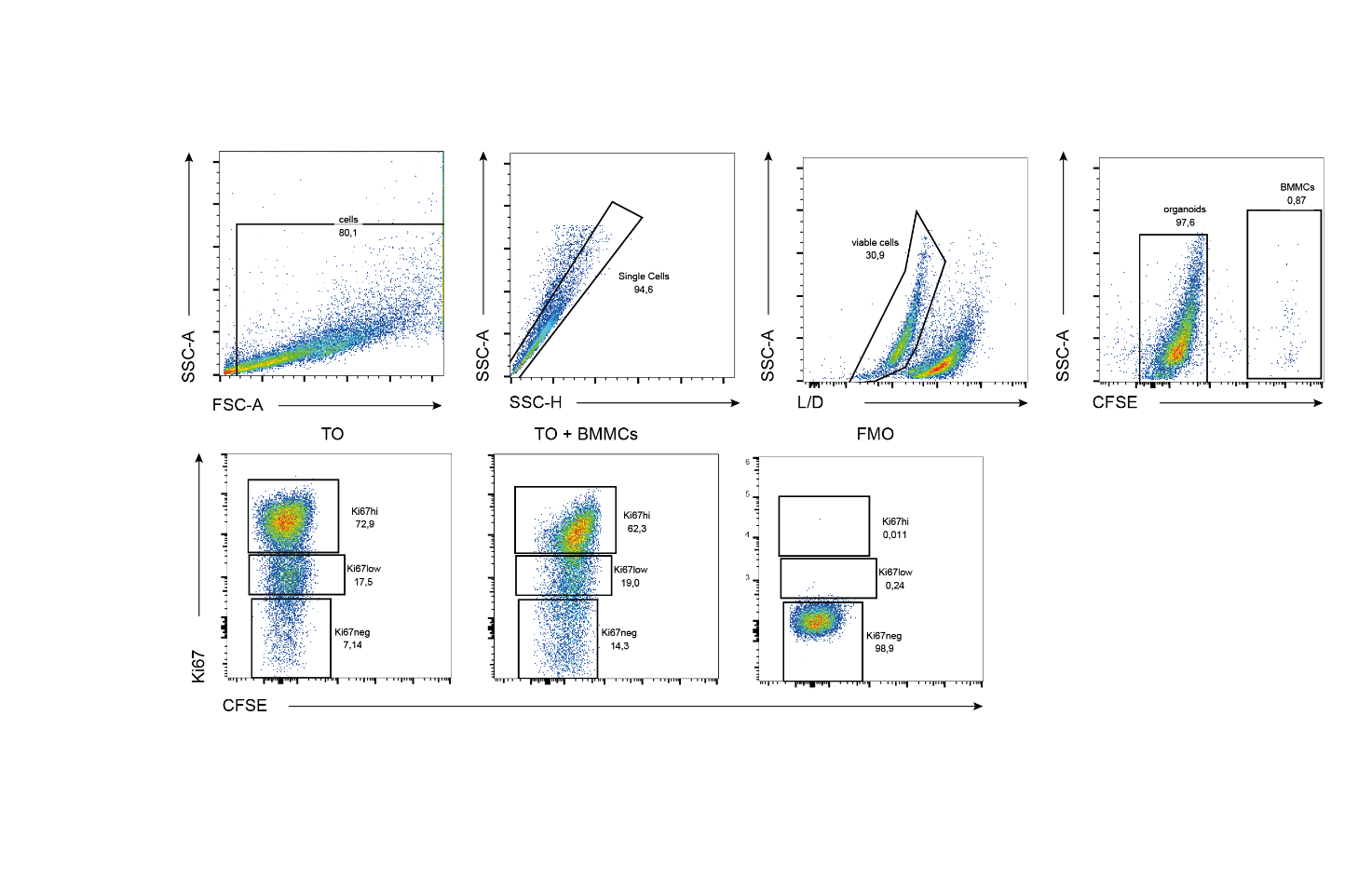 |
| **Supplemental Figure S4.** **Gating strategy for CD107 and Ki67**. **(A)** cells were selected on the FSC vs SSC plot, then only single cells (SSC-A vs SSC-H) were considered; cells negative for L/D and positive for CFSE were analyzed for CD107a expression. **(B)** Cells were selected on the FSC vs SSC plot, then only single cells (SSC-A vs SSC-H) were considered; cells negative for L/D and negative for CFSE were considered for the Ki67 analysis | |

**Supplemental Figure S5**

| **A** | **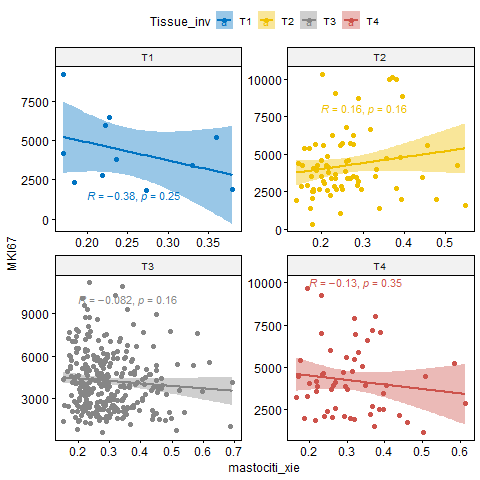** | **B** | **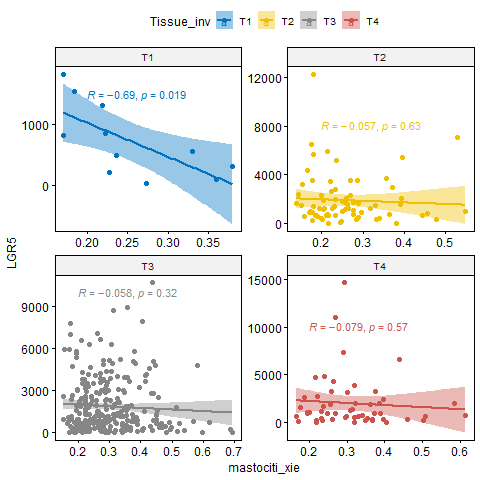** |
| --- | --- | --- | --- |
| **C** | 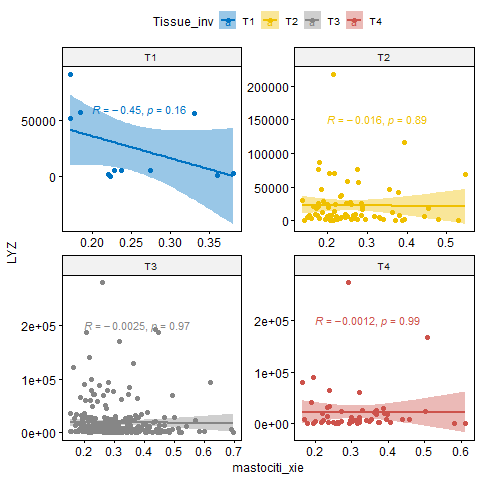 | **D** | **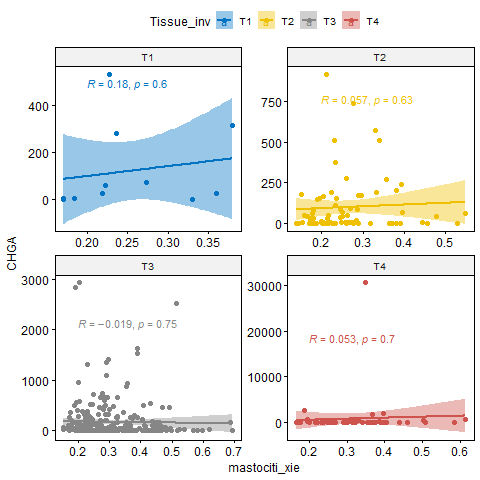** |
| **Supplemental Figure S3.** Correlation between MCs and (A) *mki67,* (B) *lgr5,* (C) *lyz* and (B) *chga* gene expression in human samples (TCGA-CRC) stratified according to tumor staging T1, T2, T3 and T4. | | | |

**SUPPLEMENTAL METHODS**

**Murine BMMCs generation and activation**

Bone marrow-derived mast cells (BMMCs) were obtained from 5-week mice by in vitro differentiation of bone marrow progenitors collected from femur and tibiae of wt or TNFα-/- mice (kindly gifted by Prof. Kollias, BSRC “Alexander Fleming”, Vari, Greece). Precursor cells were cultured in RPMI 1640 medium supplemented with 20% FBS (Sigma Aldrich), 100 U/ml Penicillin, 100 mg/ml Streptomycin, 2 mM Glutammine, 20 mM Hepes, 1X non-essential amino acids, 1mM Sodium Pyruvate, 50 mM β-mercaptoethanol (Sigma Aldrich) and 20 ng/ml IL-3 (Peprotech). All reagents were obtained from Euroclone. After 4 weeks of culture, BMMCs were monitored for FcεRI and c-Kit expression by flow cytometry and used if purity was more than 98%. For IgE-dependent activation, BMMCs were sensitized for 2 hours with 1μg/ml of dinitrophenol (DNP)-specific IgE and challenged with 100 ng/ml DNP (Sigma Aldrich).

**Metabolic analysis**

Extracellular acidification rate (ECAR) and oxygen consumption rate (OCR) were measured as indicators of aerobic glycolysis and oxidative phosphorylation (OXPHOS), respectively. Real-time measurements of oxygen consumption rate (OCR) and extracellular acidification rate (ECAR) were made using an XFe-96 Extracellular Flux Analyzer (Seahorse Bioscience). After co-culture, BMMCs were recovered from the co-cultures and plated in XFe-96 plates (Seahorse Bioscience) at the concentration of 3.5 x 105 cells/well. OCR was measured in XF media (non-buffered DMEM medium, containing 10 mM glucose, 2 mM L-glutamin, and 1 mM sodium pyruvate), under basal conditions and in response to 2 μM oligomycin. ECAR was measured in XF media in basal condition and response to 10 mM glucose and 2 μM oligomycin (all from Sigma Aldrich). ATP production was calculated as the difference between OCR before oligomycin injection and OCR after oligomycin injection. Glycolysis was calculated as the difference between ECAR before oligomycin injection and ECAR before glucose injection.

**Mouse Organoid generation**

Organoids were generated from isolated crypts of murine healthy colon and AOM/DSS derived adenomas as described in (Sato T., et al 2009) with little modifications. Crypts were resuspended in GF- basal medium (Advanced DMEM/F12 with P/S, Glutamine, Hepes, 500 nM A83-01, 100 μg/ml Primocin) and mixed with Matrigel (Corning) with 1:2 ratios and plated into pre-warmed 24 well Greiner plates. Healthy colon organoids were maintained in complete WENR medium (GF- medium supplemented with 50 ng/ml EGF, 50% Wnt conditioned medium, 20% R-Spondin conditioned medium, 10% Noggin conditioned medium) and AOM/DSS derived organoids in ENR medium (GF- medium with 50 ng/ml EGF, 20% R-Spondin conditioned medium, 10% Noggin conditioned medium).

**Proliferation rate of MC38-GFP evaluation**

MC38-GFP were seeded on 96-well plate and co-cultured with different numbers of BMMCs for 48h, then cells were fixed with PBS+3.7% PFA (Sigma-Aldrich) for 20 min, permeabilized with PBS + 0.1% TRITON X-100 (Sigma-Aldrich) for 5 min and blocked with PBS+1% FBS (Sigma-Aldrich) for 2h. The anti-GFP antibody (clone GF28R, Thermo Fisher Scientific) was diluted in PBS+1% FBS and incubated O/N at 4°, and was detected with Anti-Mouse IgG (H+L), CF™ 750 antibody (goat, Sigma-Aldrich). The fluorescence of the secondary antibody was detected with Odyssey® DLx (LicorBio).

**Western blot**

Organoid pellets were lysed in NP-40 buffer (25 mM Tris-HCl [pH 7.4], 150 mM NaCl, 1 mM EDTA, 1% NP-40, 5% glycerol, 1 mM Na3VO4, 50 mM NaF, and Complete Mini protease inhibitor cocktail (Roche) for 10 minutes on ice. Lysates were separated on SDS 10% polyacrylamide gels, blotted on nitrocellulose membrane (Amersham), and probed for Claudin-4 and actin (C4, DB Biosciences). Secondary antibodies (anti-rabbit IgG IRDye 680 and anti-mouse IgG IRDye 800) were used and detection was performed with the Odyssey CLx IR imaging system (LI-COR GmbH). Protein bands were quantified using Odyssey software (Image Studio 5.0).

**Microscopy**

BMMCs-organoids interaction was analyzed by time-lapse microscopy using the Leica AF6000LX system (DMI6000-B microscope equipped with a DFC350FX camera). Before the experiment, BMMCs were labelled with CFSE (Invitrogen) according to manufacturer’s instructions. For immunofluorescence staining, co-cultures were performed on ibiTreat μ-Slide 8 wells chambered coverslip (Ibidi). After incubation, cells were fixed with 4% PFA for 20 minutes, incubated for 20 minutes with NH4Cl 50mM, permeabilized with PBS+TritonX-100 0,5% and blocked with PBS+1% FBS at RT. Primary antibodies (Supplemental Table S1) were incubated ON at 4°C. The secondary antibody AlexaFluo488 (Thermofisher) was used 1:400 for 2 h at RT. Nuclei were stained with DAPI. Fluorescent images were collected using a laser scanning confocal microscope (LEICA TCS SP8, Leica Microsystems).

**Bioinformatic analysis**

Mast cells signature was retrieved on TCGA-CRC data samples downloaded from cBioportal (Cerami et al., 2012; de Bruijn et al., 2023; Gao et al., 2013). MCs abundance was estimated in each sample on th z-scores filtered by using *hacksig* package v 0.1.2 (under Rstudio 2023.06.0 Build 421) (Carenzo et al., 2022). *Cpa3*, *hpgds*, *tpsb2*, *tpsab1* and *ms4a2* genes were used for MCs signature as described in (Xie et al., 2023). The score obtained were then correlated with *lgr5* and *mki67* rpkm from each CRC sample stratified according to T from TNM classification.

**Table S1**. Antibodies used for cytofluorimetric assays and western blot assay.

| **Antibody (Ab)** | **Clone** |
| --- | --- |
| Anti-mouse FcεRIa-PE conjugated Ab (BioLegend) | MAR-1 |
| Anti-mouse cKit-APC conjugated Ab (Invitrogen) | ACK2 |
| Anti-mouse CD107a-APC conjugated Ab (BioLegend) | 1D4B |
| Anti-mouse Ki-67-PE conjugated Ab (BioLegend) | 16A8 |
| Anti-mouse Ezrin (Elabscience) | polyclonal |
| Anti-mouse Claudin 4 (Elabscience) | polyclonal |
| Anti-mouse Actin (BD Bioscence) | C4 |
| Anti-mouse Zonulin (ThermoFischer) | ZO1-1A12 |

**Table S2**. Murine primer sequences used for qRT-PCR analyses.

| **Gene** | **Forward** | | **Reverse** | |  |
| --- | --- | --- | --- | --- | --- |
| G3PDH | | TCAACAGCAACTCCCACTCTTCC | | ACCCTGTTGCTGTAGCCGTATTC | |
| MCPT-4 | | GGGCTGGAGCTGAGGAGATTA | | GTCAACACAAATTGGCGGGA | |
| MCPT-2 | | AAGCTCACCAAGGCCTCAAC | | ACACCACCAATAATCTCCTCAG | |
| Lgr5 | | AACATCAGTCAGCTACCCGC | | CTAGGCGCAGGGATTGAAGG | |
| ChgA | | CGATCCAGAAAGATGATGGTC | | CGGAAGCCTCTGTCTTTCC | |
| Lyz | | GTCTACAATCGTTGTGAGTTGGC | | ATAGTCGGTGCTTCGGTCTC | |
| Muc2 | | CTTCTGTGCCACCCTCGT | | TTCGGGATCTGGCTTCTT | |
| Cdh1 | | AACCCAAGCACGTATCAGGG | | GAGTGTTGGGGCATCATCA | |
| Cldn4 | | ACACGTTACTCCAGCGCTAC | | CTCTCAATGCCCCCTCAGTC | |
| Epcam | | CATTTGCTCCAAACTGGCGT | | TTGTTCTGGATCGCCCCTTC | |
| Vimentin | | TTTGCTGACCTCTCTGAGGC | | CTCCAGGGACTCGTTAGTGC | |
| TNFα | | AGGCACTCCCCCAAAAGATG | | CCATTTGGGAACTTCTCATCCC | |
| IL-6 | | ACCACTTCACAAGTCGGAGGCTTA | | TCTGCAAGTGCATCATCGTTGTTC | |
| IL-4 | | AGCCATATCCACGGATGCGACAAA | | AATATGCGAAGCACCTTGGAAGCC | |
| IL-13 | | AGGAGCTTATTGAGGAGCTGAGCA | | TGGAGATGTTGGTCAGGGAATCCA | |
| TGFβ | | TAAAGAGGTCACCCGCGTGCTGCTAAT | | ACTGCTTCCCGAATGTCTGACGTA | |

**Biobliography**

Carenzo, A., Pistore, F., Serafini, M.S., Lenoci, D., Licata, A.G., De Cecco, L., 2022. hacksig: a unified and tidy R framework to easily compute gene expression signature scores. Bioinformatics 38, 2940–2942. https://doi.org/10.1093/bioinformatics/btac161

Cerami, E., Gao, J., Dogrusoz, U., Gross, B.E., Sumer, S.O., Aksoy, B.A., Jacobsen, A., Byrne, C.J., Heuer, M.L., Larsson, E., Antipin, Y., Reva, B., Goldberg, A.P., Sander, C., Schultz, N., 2012. The cBio cancer genomics portal: an open platform for exploring multidimensional cancer genomics data. Cancer Discov 2, 401–404. https://doi.org/10.1158/2159-8290.CD-12-0095

de Bruijn, I., Kundra, R., Mastrogiacomo, B., Tran, T.N., Sikina, L., Mazor, T., Li, X., Ochoa, A., Zhao, G., Lai, B., Abeshouse, A., Baiceanu, D., Ciftci, E., Dogrusoz, U., Dufilie, A., Erkoc, Z., Garcia Lara, E., Fu, Z., Gross, B., Haynes, C., Heath, A., Higgins, D., Jagannathan, P., Kalletla, K., Kumari, P., Lindsay, J., Lisman, A., Leenknegt, B., Lukasse, P., Madela, D., Madupuri, R., van Nierop, P., Plantalech, O., Quach, J., Resnick, A.C., Rodenburg, S.Y.A., Satravada, B.A., Schaeffer, F., Sheridan, R., Singh, J., Sirohi, R., Sumer, S.O., van Hagen, S., Wang, A., Wilson, M., Zhang, H., Zhu, K., Rusk, N., Brown, S., Lavery, J.A., Panageas, K.S., Rudolph, J.E., LeNoue-Newton, M.L., Warner, J.L., Guo, X., Hunter-Zinck, H., Yu, T.V., Pilai, S., Nichols, C., Gardos, S.M., Philip, J., AACR Project GENIE BPC Core Team, AACR Project GENIE Consortium, Kehl, K.L., Riely, G.J., Schrag, D., Lee, J., Fiandalo, M.V., Sweeney, S.M., Pugh, T.J., Sander, C., Cerami, E., Gao, J., Schultz, N., 2023. Analysis and Visualization of Longitudinal Genomic and Clinical Data from the AACR Project GENIE Biopharma Collaborative in cBioPortal. Cancer Res 83, 3861–3867. https://doi.org/10.1158/0008-5472.CAN-23-0816

Sato T, Vries RG, Snippert HJ, Van De Wetering M, Barker N, Stange DE, et al. Single Lgr5 stem cells build crypt-villus structures in vitro without a mesenchymal niche. Nature. Nature Publishing Group; 2009;459:262–5.

Gao, J., Aksoy, B.A., Dogrusoz, U., Dresdner, G., Gross, B., Sumer, S.O., Sun, Y., Jacobsen, A., Sinha, R., Larsson, E., Cerami, E., Sander, C., Schultz, N., 2013. Integrative analysis of complex cancer genomics and clinical profiles using the cBioPortal. Sci Signal 6, pl1. https://doi.org/10.1126/scisignal.2004088

Xie, Z., Niu, L., Zheng, G., Du, K., Dai, S., Li, R., Dan, H., Duan, L., Wu, H., Ren, G., Dou, X., Feng, F., Zhang, J., Zheng, J., 2023. Single-cell analysis unveils activation of mast cells in colorectal cancer microenvironment. Cell & Bioscience 13, 217. <https://doi.org/10.1186/s13578-023-01144-x>
